# The Celluloid Base

**Published:** 1872-03

**Authors:** Geo. W. Tripp


### ARTICLE IX.

### *The Celluloid Base.*

BY GEO. W. TRIPP.

Mr. Editor,—In a communication of mine which appeared in the January number of the *Dental Cosmos*, I remarked that I held myself ready to impart instructions to such of the profession as might be unacquainted with the processes of working the celluloid base successfully. I admit that this offer was put forth to induce my brethren of the profession to abandon the use of rubber, and I foresaw that the proposition would devolve on me replies to letters from those disposed to avail themselves of such offer; but I hardly anticipated such a shower of letters as the mails daily pour in on me, which I find it impracticable to answer individually. I therefore avail myself of the pages of the *Dental Cosmos* to reply to all who have done me the honor to take me at my word.

For myself, I am willing to adopt celluloid *to the entire exclusion of rubber* for both new work and for repairing old plates, made of either celluloid or rubber, on the satisfac tory assurance given me that the late improvements upon the earlier processes of producing the celluloid base secures a hardness, toughness, solidity, and permanency of form that compares favorably with rubber. The desideratum of avoiding *shrinking of the plate* is claimed to have been attained by the successful hardening, toughening, and solidifying process in the manufacture of the celluloid base; and with this (to me) satisfactory assurance, I have no hesitation in recommending it to my patrons as indeed preferable to rubber, in view of the demonstrable fact that the rubber base contains properties in its composition prejudicial to health.

In writing for publication, I am necessarily restricted in space, and can present but a few of the more important and prominent rules or guides to its successful use.

1st. *It is to be worked in oil*, sufficient heat not being attainable with water in an open vessel; the highest temperature which can thus be reached being 212°, whereas the working of the celluloid requires 320°.

2d. *To mend a celluloid plate*, cut away the plate as required; then wet celluloid filings or scrapings with ether and alcohol (which, combined of two thirds ether and one-third alcohol, are a solvent of celluloid), and when in a tacky or thick plastic state, slightly dress the parts with it, which insures a more ready union. This done, pour your solvent into a small glass vessel, closing the top with the palm of the hand or otherwise, in order to prevent the escape of the ether. Into this put the piece of celluloid to be used, allowing it to remain immersed a few minutes; until the surface only becomes somewhat plastic; then take it out and place it in position, and heat in oil to a temperature of 320°, by the thermometer. On removing the cast from the oil, care must be taken to *cool it thoroughly before opening the flask* for cutting out the plate, in order to obviate any warping or change of form. The parts will be found united perfectly.

The most delicate thin gums need never be broken in the use of celluloid. As a safe precaution to insure firmness of the teeth in position, it will be well to champer the tops of the gums, so that the celluloid may be left intact over them when finished. Raise the heat to 260°; then turn down a little with the finger to a point of *slight* resistance, being careful not to force it much until you reach 300°, but when 320° are indicated, *down with it.* No fear need be apprehended of a softening of the cast from heating; it only becomes the harder. No solutions are needed to harden the cast. Any imperfection found in a plate on removing it from the flask may be readily repaired and made complete by using a piece of celluloid (first moistening its surface and that of the imperfect portion of the plate with the solvent), by rubbing and pressing it into position with the fingers, leaving it to be worked down when dry; or, the filings or scrapings may be moistened with the solvent and applied with a spatula. The former is perhaps preferable. In either case, however, a reheating is unnecessary.

It may be esteemed worthy of mention, that when a celluloid base is found to be not in acceptable form for the cast to be used, it may readily be adapted to it by immersing in boiling water, when it can be contracted or distended to meet the want.

The question is asked, is celluloid as strong as rubber? I reply, I have never known one to break. When in the mouth it becomes more elastic from the warmth imparted to it; hence, adapting itself more perfectly, it is less liable to break in using. Patrons should be advised to wear their plates on retiring for the night, thus avoid any risk of contraction or shrinking. Should any contraction or shrinking take place, it may easily be relieved by scraping off a little at the proper points.

Again it is asked, can a new block of teeth be attached with celluloid to a rubber plate? I answer, dovetail, drill holes through plate, countersinking on opposite side (just as is done when rubber is used to repair with); place celluloid in position, and heat up in oil, pressing together as in making a new celluloid plate.

It is further asked, can a plate be used a second time in case of being obliged to work it over? I answer, it can be done successfully by wetting the parts with the solvent. If teeth are to be substituted, remove the old ones, and proceed with the old plate as with a new one. If there be any broken parts to repair, soften your filings or scrapings of celluloid with the solvent, and apply the same to the broken parts, avoiding a too liberal use of the plastic mass.

In using scraps of celluloid for repairing a plate, soften their surfaces in the solvent (as before stated), when the parts will unite one with another perfectly, and as firmly as an original base or plate. It need not necessarily be in one entire piece to insure strength. A good hard cast is of primary importance.—*Dental Cosmos.*

